# Evidence-based guidelines and decision support services: a discussion and evaluation in triple assessment of suspected breast cancer

**DOI:** 10.1038/sj.bjc.6603470

**Published:** 2006-11-21

**Authors:** V Patkar, C Hurt, R Steele, S Love, A Purushotham, M Williams, R Thomson, J Fox

**Affiliations:** 1 Advanced Computation Laboratory, Cancer Research UK, London WC2A 3PX, UK; 2 Medical Statistic Group, Cancer Research UK, Wolfson College Annexe, Linton Road, OX2 6UD, UK; 3 Kings College London, Department of academic Oncology, Guy's – St Thomas's NHS Foundation Trust, London SE1 9RT, UK

**Keywords:** computerised decision support, clinical practice guidelines

## Abstract

Widespread health service goals to improve consistency and safety in patient care have prompted considerable investment in the development of evidence-based clinical guidelines. Computerised decision support (CDS) systems have been proposed as a means to implement guidelines in practice. This paper discusses the general concept in oncology and presents an evaluation of a CDS system to support triple assessment (TA) in breast cancer care. Balanced-block crossover experiment and questionnaire study. One stop clinic for symptomatic breast patients. Twenty-four practising breast clinicians from United Kingdom National Health Service hospitals. A web-based CDS system. Clinicians made significantly more deviations from guideline recommendations without decision support (60 out of 120 errors without CDS; 16 out of 120 errors with CDS, *P*<0.001). Ignoring minor deviations, 16 potentially critical errors arose in the no-decision-support arm of the trial compared with just one (*P*=0.001) when decision support was available. Opinions of participating clinicians towards the CDS tool became more positive after they had used it (*P*<0.025). The use of decision support capabilities in TA may yield significant measurable benefits for quality and safety of patient care. This is an important option for improving compliance with evidence-based practice guidelines.

One of the most consistent findings in health services research is the gap between evidence and practice (Grol and Grimshaw, 2003). Consistent, safe, evidence-based health care has become a major goal of many health-care systems, in developed countries in particular, but is not always achieved (Kohn *et al*, 1999; Corrigan *et al*, 2001). In the UK, it has been found that about 850 000 medical errors occur in National Health Service hospitals every year, resulting in some 40 000 deaths (Aylin *et al*, 2004) and other consequences. In 2001, the UK Audit Commission's report on NHS cancer care in England and Wales showed significant variation at all stages of cancer care, including the criteria used by general practitioners to refer patients, the diagnostic tests ordered and the type of surgery, chemotherapy, and radiotherapy offered.

Such findings have fuelled worldwide interest in developing clinical practice guidelines (CPGs) on the basis that they can be expected to help improve quality of care by disseminating research results and evidence-based practice more effectively. Many studies have shown that CPGs can improve the quality of care (Cabana *et al*, 1999; James and Hammond, 2000; Grimshaw *et al*, 2002). A CPG is a ‘systematically developed statement to assist practitioner and patient decisions about appropriate health care for specific clinical circumstances’ (Field and Lohr, 1990 Institute of Medicine).

In a special issue of *BJC* on *Clinical Practice Guidelines* for cancer care (2001) Fervers, Hardy, and Philip introduce the SOR (Standards, Options, and Recommendations) guidelines project of the French Federation of Cancer Centers. Standards, Options, and Recommendation is a major ongoing national project (http://www.fnclcc.fr/) whose goal is to develop a methodology for the progressive creation and maintenance of ‘CPGs for the initial management of cancer in adults and children, for supportive care and control of symptoms in cancer patients and for the standardisation of ‘good clinical practice’ throughout the various disciplines involved in cancer care. It has also undertaken the developments of CPGs specifically for nursing and paramedical staff, as well as the provision of evidence-based information for patients. (Fervers *et al*, 2001).

The end result of the SOR development process is a document (paper or electronic). A SOR document typically contains a collection of ‘clinical algorithms’ to be consulted in appropriate situations together with a succinct summary, rationale, and evidence for the Standards, Options and criteria for Recommendations covered by the algorithms. The *BJC* special issue includes articles that describe a diverse set of 14 representative SOR guidelines, covering common and less common cancers, developed by many specialist groups. It documents 45 complete CPGs, involving contributions from some 1700 doctors, pharmacists, and biologists.

By any standards this is an impressive body of work, which on the face of it could well justify the effort and resources that Fervers *et al* (2001) emphasise is needed. The SOR programme has attracted considerable international attention and extensive collaborations with specialist cancer groups in the Canada, the USA, and UK. Assuming that the methodology is rigorously followed we may reasonably assume that the content of a SOR CPG can be trusted, and assuming that the CPG is maintained properly as new research is published such guidelines can be an important continuing resource, facilitating discussion and new research as well as specific clinical guidance.

There are, however, significant issues about the practical use of practice guidelines, both SOR guidelines and other approaches. Fervers *et al* (2001) themselves identify a number of obstacles, including the problems of dissemination and continuing maintenance of the guideline content, which they refer to as the ‘aftercare’ problem. The question raised in this paper goes further, asking about the use of and compliance with such guidelines in the clinic. Although there is evidence of clinical value there are also grounds for concern that the great potential value of the enormous effort that goes into creating the guidelines may not be matched by the level of adherence to them in practice (Bloom *et al*, 2004). Furthermore a systematic review has found that traditional paper-based dissemination of guidelines are relatively ineffective in changing the behaviour of health-care professionals (Freemantle *et al*, 1996).

Because of such concerns about the penetration of evidence-based guidelines into routine clinical practice informaticians have investigated techniques for bringing CPGs to the point of care in a more useful form than documentary reminders and algorithms. One prominent development is computerised Clinical Decision Support Systems (CDSSs) which add value to conventional guidelines by delivering options and recommendations in the form of *patient-specific suggestions*. A recent systematic review of CDSSs suggested that systems for disease management improve practitioner compliance with guidelines: in the majority of randomised trials (23 out of 37 studies, i.e. 62%) and demonstrated a positive impact (Garg *et al*, 2005). Our group has developed a series of CDSSs for use in cancer care with very promising results, including a system for genetic risk assessment based on family history (Emery *et al*, 2000), detection of abnormalities in mammograms (Taylor *et al*, 1999) and chemotherapy dosage decisions in paediatric ALL (Bury *et al*, 2005).

Most decision support techniques have tended to focus on isolated decision nodes in the care process, for example, drug dosing (Manotti *et al*, 2001) or ECG analysis (Selker *et al*, 1998), rather than on a ‘patient journey’ as an integrated and coordinated whole. Our work on the CREDO project (Fox *et al*, 2006) is designed to support the entire journey of breast cancer patients, from initial presentation and diagnosis through to treatment and follow-up. As part of the CREDO project we have developed a formal model of a care pathway for the management of women with breast cancer, or at risk of developing breast cancer, as a foundation for the design of the decision support and other services based on published CPGs and other evidence-based sources. The model shows that there may be as many as 65 separate decision points in the breast cancer journey where if best evidence-based practice is not complied with there is significant potential for patient harm, or at least failure to achieve the best outcome.

There were two purposes of the present study. First, to investigate whether decision support technology can significantly enhance the compliance of breast clinicians with best practice as defined by evidence-based guidelines. Second, to determine the benefits of a specific approach to decision support in CREDO. The focus of the study is the initial (triple) assessment of patients referred to breast clinics with symptoms of possible cancer. In the United Kingdom triple assessment (TA) clinics are carried out by the members of a multidisciplinary team to diagnose and manage symptomatic patients. Such clinics involve decision-making based on clinical examination, radiological, and pathological investigations conducted in one session with the aim of speeding up diagnosis and treatment. The Triple Assessment Decision Support system (TADS) is designed to assist at four decision points (family history and genetic risk assessment, selection of imaging and biopsy modalities, and final management decision).

## MATERIALS AND METHODS

### PROforma tools for modelling clinical guidelines and pathways

The TADS system was constructed using the PRO*forma* guideline and workflow modelling language (Fox and Das, 2000) and the Tallis process modelling system developed by Cancer Research UK (www.acl.icnet.uk/TallisTrainin g).

The key difference from the SOR approach is that the PRO*forma* model can be *executed* by a computer and displayed at the clinical point of care using, for example, a web browser. Tallis provides many ways of delivering decision support but a straightforward implementation would provide electronic data forms for recording patient data, automatic scheduling of clinical tasks and display of SOR standards and, if required, making patient-specific recommendations for diagnostic or therapeutic options according to patient data and clinical circumstances. In this way a PRO*forma* service can deliver conventional guidelines such as SOR guidelines while adding patient-specific decision support and many other data and knowledge management services.

### TA mode

**Figure 1:**
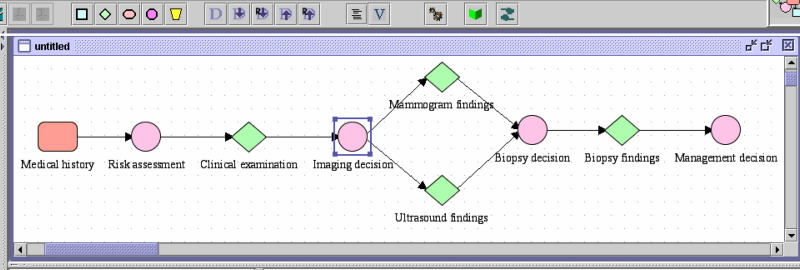
Tallis representation of TA workflow showing the main plan. The decision nodes represented by circles are embedded at various points in the workflow.

TADS was designed to support a breast clinician taking a patient through the TA clinic. Figure 1 shows the Tallis representation of TA workflow, which involves abstracting data from family history, medical history, clinical examination of a patient and making decisions about risks, and diagnostic interventions and management.

1. Four key inter-dependent decision points were identified in the TA workflow:
2. Genetic risk assessment: low, medium or high (taken as part of the clinical history plan)
3. Radiological investigations to perform: mammogram, ultrasound, both or none
4. Biopsy method to perform: (FNA, core biopsy, and other investigations)
5. Management decision: whether to refer the patient to a multidisciplinary team and/or to geneticist, to discharge or to follow-up (high-risk surveillance).

### Designing the medical knowledge base

Evidence-based guidelines (see Table 1) were used to define the logical reasoning about patient data as a set of arguments for and against each option, for each of the four decisions. All selected CPGs scored high (overall score of >60% in all domains) on a 23-point AGREE scale (The AGREE collaboration, 2003) suggesting their high rigour. An expert panel was formed comprising four senior practising consultants from four relevant disciplines (surgery, radiology, pathology, and genetics). The panel reviewed the knowledge base for its accuracy in encoding the evidence-based guidelines and consensus was achieved.

### Hypothetical cases

We developed 15 hypothetical cases, designed to cover a range of risk levels and clinical scenarios. Cases were adapted from a larger number of real cases referred to the Guy's Hospital TA clinic over a period of 6 months. We specified all the patient data that would be required for taking all four key decisions, such as complete medical and family history, complete examination findings, and test results. The set of 15 cases was reviewed by the expert panel for the adequacy of data, as well as for internal consistency and generalisability.

A knowledge base of 125 evidence-based arguments/facts derived from the guidelines allowed TADS to mimic exactly the expert panel's recommendations for all decisions in the test set of 15 cases. From these 15 cases, three sets of five simulated cases were established, each set taking a variety of scenarios.

### Design of the study

We performed a crossover experiment with balanced block design in which participating clinicians were asked to address the cases, with and without decision support. A total of 36 breast clinicians were opportunistically sampled from the population of breast cancer clinicians who routinely conduct TA clinics in the south east of the England. In all, 24 agreed to participate in the trial.

**Figure 2:**
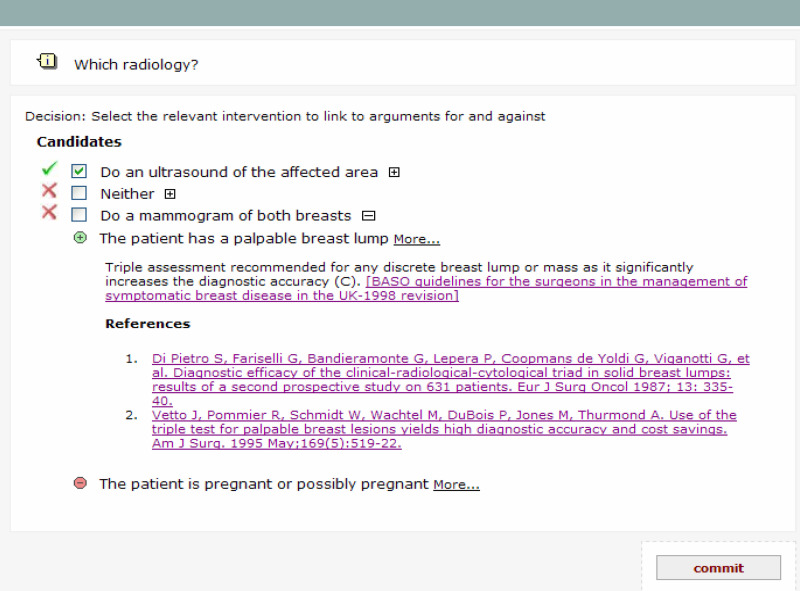
TADS screen with decision support enabled, showing decision options for the imaging for one case, to be taken after medical history and examination. The system recommends an ultrasound scan but recommends against mammography and against doing nothing. For the decision option ‘Do a mammogram of both breasts’, arguments for and against have been expanded to show the justifying evidence (an option available to the clinician for all decisions, options and arguments). Links are provided to the relevant supporting literature, which can be accessed by the user if required (e.g. from PubMed).

For the trial, TADS was designed to run in two ‘modes’ with decision support either enabled or disabled. In both modes, the patient information required to take a decision is displayed on the computer screen at the decision point. In decision support enabled mode (DS+), recommended decision options are highlighted by green ticks; non-recommended options are marked by red crosses. The user can also see the medical reasons for and against each option, along with hyperlinks to the referring guideline and underlying literature evidence (see Figure 2). However, the user always has the freedom to override system recommendations.

**Figure 3:**
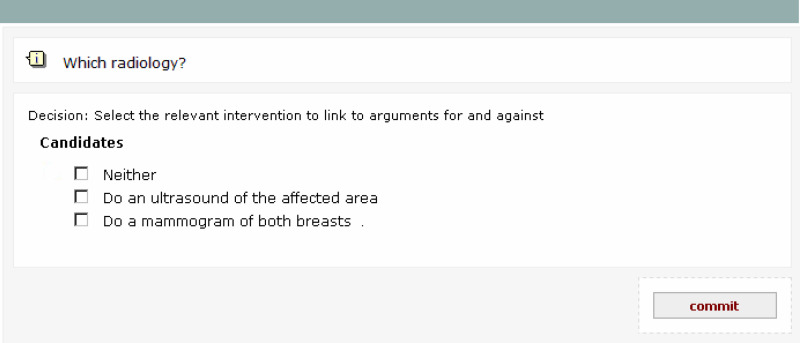
TADS screen with decision support disabled, showing options for imaging after medical history and examination have been presented.

In decision support disabled (DS−) mode, the system displays the list of options without highlighting recommendations (see Figure 3). In both modes, the system anonymously captures and keeps track of the options selected by the user at each decision point, together with a record of what its own recommendation would have been in the DS+ mode.

Each participating clinician was assigned to address two sets of cases: one set with and one set without decision support. Assignment as to whether decision support was to be made available for the first or second set was randomly balanced to control for any learning effect. To control for differences in the difficulty of the case sets, the three sets of five cases were also balanced so that each set was addressed the same number of times in each arm of the trial. We refer to each patient being taken through the four decisions by one subject as a ‘patient journey’, so in total 120 patient journeys (24 clinicians going through a set of five patients) were made with decision support and 120 without.

Sessions were conducted using a laptop computer in offices/clinics in the various hospitals in which the participating clinicians worked. All subjects were familiarised with the system using a prepared training script. Throughout each session, subjects had access to each patient's data on paper as well as via TADS web pages.

### Statistical method

After the experiment was completed, the decisions made by each clinician for each patient case were compared to guideline recommendations as determined by our expert panel. Each ‘patient journey’ was categorised as either ‘with deviations’, or ‘without deviation’.

Deviations were empirically categorised by the expert panel as follows:

- Minor or non-critical deviations that arguably would not result in direct patient harm.
- Critical deviations that could potentially result in patient harm.

A further subgroup of critical deviations was identified as irretrievable critical deviations, where a patient completed the journey and was discharged. In practice, such errors would typically not be spotted or rectified by other members of the team.

The analysis of the patient journeys was carried out on a per clinician basis. The number of patient journeys that contained errors or deviations in both decision support and no decision support arms were compared using a Wilcoxon signed rank test. We had 80% power to find a 15% significant difference (*P*=0.05).

## RESULTS

### Population characteristics

The majority of our study population were male consultant breast surgeons with intermediate computing skills. On average, they were 42.9 years old with 9.4 years experience in the speciality of breast cancer and had been conducting 1.8 TA clinics a week for 6.5 years.

The average time taken by each clinician to complete 10 patient journeys was 37.2 min ranging from 24 to 61 min.

### Analysis of deviations

Sixty out of 120 patient journeys undertaken in the DS− condition included at least one deviation (see Table 2), compared with only 16 out of 120 supporting the DS+ condition (*P*<0.001). Out of a total of 60 deviations in the DS− arm of the trial, 16 were identified as potentially critical, compared to only one in the DS+ arm (*P*=0.001). In all, 10 out of 120 patient journeys without decision support involved at least one deviation that was irretrievable and potentially critical, compared to only one out of 120 with decision support (*P*=0.02).

Examples of deviations or errors recorded in the study

Minor or non-critical deviations

- Requesting ultrasound as a screening tool in the absence of any localised abnormality
- Not requesting ultrasound for breast mass
- Overestimating familial breast cancer risk
- Underestimating familial breast cancer risk
- Unnecessary referral to a geneticist (of a low-risk patient)
- Unnecessary referral to multidisciplinary team.

Potentially critical deviations

- Failing to perform FNA/biopsy in the event of a localised clinical abnormality and normal imaging.
- Not requesting ultrasound when a mammogram showed localised density/mass.
- Failing to carry out a repeat biopsy when the FNA report for a breast lump was inconclusive.
- Failing to request a mammogram for symptomatic women over 35 years with high genetic risk.
- Failing to request a mammogram for a pregnant woman when clinical examination and ultrasound were both highly suggestive of malignancy.
- Not requesting a mammogram for a symptomatic woman over 30 years with high risk owing to mantle radiotherapy in childhood.

### Questionnaires

At the end of the trial, we conducted a questionnaire study with participants to learn more about their thoughts on the system. An opinion on a five-point Likert scale, to the most basic statement: ‘patient care in TA would benefit from computerised decision support’, was obtained from participants both before and after use of the system. To investigate the change in opinion we used the Fleiss Everitt simplification of the Stuart Maxwell test for matched pairs (Fleiss, 1981) to look for a change in distribution of opinion and then checked for systematic differences using the McNemar test (McNemar, 1947). Overall, 10 clinicians maintained the same opinion of TADS both before and after the experiment; 11 became more convinced of its benefit and two became one category less convinced (Table 3).

There was a highly significant difference in the change in distribution of opinion (matched pair, 3 d.f., *χ*^2^=10.26, and *P*<0.01). One person who disagreed with the statement before did not change his mind afterwards, but of the 16 who were undecided before, 10 changed their minds to agree with the statement (2 d.f., *χ*^2^=8.1, and *P*<0.025).

## SUMMARY AND DISCUSSION

The results obtained in this study suggest that a decision support tool like TADS that supports multiple decisions in a care pathway can significantly reduce deviations from best practice. Though the majority of deviations observed were empirically characterised as minor, those that involved unjustified referrals and unnecessary tests could have had an adverse impact on resource usage and also costs. After excluding minor deviations, differences remained significant even for the small number of irretrievable deviations that were potentially critical. There was an overall significant positive shift in the opinions of participating clinicians towards TADS after they had used it.

This study forms part of the CREDO project, which is investigating whether evidence-based decision support technology can help to improve quality and safety of decision making in cancer care, and in particular whether PRO*forma* technology can provide comprehensive support for the real cancer journey, taking breast cancer as a model. Previous studies have looked at use of CDSS systems in genetic risk assessment (for breast and ovarian cancer) and detection and interpretation of abnormalities in mammograms, with promising results. This study represents a significant advance on these studies by considering TA, which is an important and more complex component of breast cancer management. Where earlier studies focused on a single decision, TADS includes four interdependent decisions as well as management of the clinical workflow required to provide a TA service.

The study adds to a growing body of evidence that decision support systems have significant value, both because of the focus in cancer, where there is still relatively little specific evidence about use of CDSSs and because almost all CDSS results, which have been systematically reviewed, have only considered individual decisions. The results give increased confidence that the CREDO objective of supporting the whole cancer journey is technically practical, that it could provide measurable benefits, and that such services will be acceptable to cancer professionals.

The results of the study may, however, need to be treated with some caution.

First, the study was conducted with simulated patients; the simulations were based on real cases but for this systematic comparison of decision making with and without decision support ethical and practical constraints forced us to use computer-based presentation of patient data rather than real patients. In previous research, however, simulated case scenarios have been shown to be good predictors of clinical performance (O'Hagan *et al*, 1986). Our control group also had patient data displayed to them through web pages, but paperless records have been shown to be more understandable than paper-based records (Hippisley-Cox *et al*, 2003) and subjects in the control group were forced to consider all pertinent patient data and make all decisions in our simulation. Continued exposure to CDSSs may improve clinical performance still further as users get more experienced in their use. In other studies we have asked actors to play the role of patients, with similar results in terms of improved decision-making. Using trained actors to role-play patients is an accepted technique which accurately predicts behaviour in real clinical settings but is still a very demanding evaluation technique in complex, multi-decision settings like TA. Given the generally positive results reported in studies of CDSSs and in the absence of a reason to suggest that our results could be an artefact of the simulation we are inclined to accept the findings. For the present, however, the results must be treated as indicative rather than definitive until a full RCT has been completed.

Second, the simulated cases were deliberately more varied than one would find in a typical TA clinic. One of our aims was to test the effect of the software in differing scenarios. Consequently, more diverse cases than one would typically find in 15 cases chosen at random were presented. It may also be argued that our study sample of doctors was not representative but consisted of a self-selecting group of sufficiently computer literate clinicians. However, we see no reason to doubt the apparently substantial benefits of decision support purely on this variety; rather the reverse. Indeed only six clinicians thought that their decision-making in TA would benefit from decision support before they participated in the study while after participation this figure increased to 16.

The SOR methodology is a particularly relevant context in which to consider the extension of CPGs to include decision support capabilities because it has been developed primarily with cancer applications in mind, and because the participants in the SOR programme have created a great deal of oncological content which might be enhanced with decision support functions. The PRO*forma* approach seems well suited to adding value to SOR CPGs because of the straightforward mapping between SORs and the PRO*forma* decision model. This model is based on the automated construction of arguments for and against decision options, where each argument has an explicit logical justification together with an evidence-based grounding in published research. PRO*forma* can also be used to add value to other document-centric guideline systems like GEM (Shiffman *et al*, 2004) but the SOR method is attractive because of the rigorous approach taken to the creation of a large set of cancer CPGs. We may also note that PRO*forma* can offer a variety of additional services over and above decision making which are relevant to routine cancer care, including treatment planning and the management of clinical trials and capture of patient data with electronic CRFs.

## Figures and Tables

**Table 1:** Guidelines used in the DSS

**Specialty**	**Guidelines**	**AGREE score**
Genetic risk assessment	NICE: Familial breast cancer guideline	88
Diagnosis	SIGN: Management of breast cancer in women	82
	BASO: Guidelines for surgeons in the management of symptomatic breast disease in the UK	69
	NCCN: Breast Cancer Screening and Diagnosis guidelines	75
Imaging	ACR: Appropriateness Criteria	70
	NHSBSP guidelines: Breast cancer screening assessment (Pub. 49)	63
Pathology	NHSBSP guidelines: Non-operative diagnostic procedures and reporting in breast cancer screening (Pub. 50)	63

DSS=Decision Support System.

The table includes overall AGREE score for each clinical practice guideline out of a maximum score of 92.

**Table 2:** Analysis of deviations in decision support and no-decision support arms

	**Without decision support (Total 120 patient journeys)**		**With decision support (Total 120 patient journeys)**		
**Type of deviation**	**Patient journey with at least one deviation of given type**	**Patient journey without any deviation of given type**	**Patient journey with at least one deviation of given type**	**Patient journey without any deviation of given type**	***P* value by Fisher's exact**
All deviations	60	60	16	104	<0.001
Potentially critical deviations	16	104	1	119	<0.001
Potentially critical irretrievable deviations	10	110	1	119	0.01

**Table 3:** Responses to the statement: ‘patient care in triple assessment would benefit from computerised decision support’

	**Before using TADS**	**After using TADS system**
Strongly agree	3	3
Agree	3	13
Undecided	16	6
Disagree	1	2
Strongly disagree	1	0
Total	24	24

TADS=The Triple Assessment Decision Support System.

Ten clinicians maintained the same opinion of TADS before and after; 11 became more convinced of its benefit; two became one category less convinced of its benefit.
